# Etiology and Management of Treatment-Resistant Hypertension in African American Adults ≥18 Years: A Literature Review

**DOI:** 10.7759/cureus.29566

**Published:** 2022-09-25

**Authors:** Lilian O Odion-Omonhimin, Farirai M Marwizi, Mimidoo Chive, Nmachi B Obasi, Abidemi O Akinrinmade, Vivien O Obitulata-Ugwu, Folami Victor, Nkechi B Obijiofor

**Affiliations:** 1 Medicine and Surgery, University of Benin, Benin, NGA; 2 Cardiology, Universitatea de Medicina si Farmacia din Timisoara, Timisoara, ROU; 3 Infectious Disease, Richmond Gabriel University, Arnos Vale, VCT; 4 Cardiology, Rivers State University Teaching Hospital, Port Harcourt, NGA; 5 Medicine and Surgery, Benjamin S. Carson School of Medicine, Babcock University, Ilishan-Remo, NGA; 6 United States Medical Licensing Examination (USMLE) Education, Kaplan Medical Prep, Manhattan, USA; 7 College of Medicine, University of Nigeria, Enugu, NGA; 8 College of Medicine, Grodno state medical University, Grodno, BLR; 9 Medicine and Surgery, Nnamdi Azikiwe University, Awka, NGA

**Keywords:** blood pressure, prevalence, management, etiology, african americans, treatment-resistant hypertension

## Abstract

Treatment-resistant hypertension (TRH) is defined as blood pressure levels that remain above the therapeutic goal despite concurrent use of three or more antihypertensive medications taken at maximally tolerated doses, one of which should be a diuretic. Additionally, individuals on four or more antihypertensive agents regardless of blood pressure are also considered to have TRH. Amongst people diagnosed with TRH, African American adults face a huge management gap, resulting in increased cardiovascular disease risk. The primary objective of this review was to identify the commonly encountered etiologies and extensively discuss the current management strategies of TRH with a particular focus on African Americans. Relevant studies were identified by analyzing scientific databases and journals such as PubMed, Cochrane, MEDLINE, Cureus, and American Heart Association (AHA). The studies identified and examined common causes of TRH, describing their pathophysiology and highlighting different treatment options for the respective etiologies. The most prevalent etiologies of TRH amongst African Americans were chronic kidney disease (CKD), renal artery stenosis (RAS), fibromuscular dysplasia, obstructive sleep apnea (OSA), endocrine causes (Conn syndrome, Cushing syndrome, etc.), sympathetic nervous system overactivity, lifestyle factors, inaccurate blood pressure measurement, and inappropriate treatment. Of the etiologies reviewed, OSA, lifestyle factors, and CKD exhibited a striking prevalence among the subpopulation studied. Unfortunately, there was a paucity of articles addressing this topic amongst African Americans, and therefore there was not a substantial appreciation of the prevalence of some of the identified etiologies in the population of interest. Thorough diagnostic testing for associated or underlying conditions provides a basis for successful management. This review brought to the fore the need for doctors and patients to collaborate in order to improve TRH management and help patients lead healthier lives.

## Introduction and background

Treatment-resistant hypertension (TRH) is a frequently encountered clinical challenge that primary care clinicians, specialists, and patients face [1]. According to the American Heart Association (AHA), TRH refers to blood pressure that remains above the therapeutic goal despite the concurrent use of three or more antihypertensive medications taken at maximally tolerated doses, one of which should be a diuretic. Calcium channel blockers (CCBs), angiotensin-converting enzyme (ACE) inhibitors, and angiotensin receptor blockers (ARBs) are also commonly used classes. Interestingly, another definition of this phenomenon is well-controlled hypertension, which requires four or more antihypertensive agents to achieve the therapeutic target [1-3].

Many risk factors are associated with TRH, but older age, African American race, diabetes mellitus, obesity, and chronic kidney disease (CKD) are amongst the most common. Additionally, specific underlying causes play a pivotal role in TRH pathophysiology. Examples include obstructive sleep apnea (OSA), Conn syndrome, and renovascular diseases [4].

To be effective, treatment strategies for TRH must address three fundamental principles: availability, affordability, and the ability to be adapted to different healthcare settings. Practically, treatment modalities may not always fulfill the aforementioned factors within a given clinical scenario [5]. This review explores different treatment approaches to TRH, specifically in African American adults due to its higher prevalence amongst people of this race. By achieving an individual therapeutic blood pressure target in African Americans, 400,000 cardiovascular events could be prevented annually [6].

## Review

Chronic kidney disease

To better understand the correlation between TRH and CKD, it is essential to illustrate prior situations leading to CKD. As per the guidelines outlined by the Kidney Disease Outcome Quality Initiative of the National Kidney Foundation (NKF), CKD refers to kidney damage or an estimated glomerular filtration rate (eGFR) lower than <60 mL/min/1.73 m² for at least three months [7]. The precipitating factors associated with TRH include older age, lower GFR (<60 mL/min/1.73 m²), higher albuminuria and body mass index, male sex, African origin, and presence of diabetes mellitus [8-9]. Notably, long-standing CKD and type 2 diabetes are strongly associated with TRH [10]. A study on African American adults showed a 30% incidence of TRH in patients with CKD during their follow-up, while a chronic renal insufficiency cohort study showed that the prevalence of TRH was higher in patients with lower eGFR [9]. CKD predisposes to TRH by continued and persistent stimulation of the renin-angiotensin-aldosterone system (RAAS) and sympathetic nervous system, which results in high sodium retention and increased peripheral resistance [9]. There are five stages of CKD and these stages with their associating eGFR include: stage 1 (>90 ml/min/1.73 m²), stage 2 (60-89ml/min/1.73 m²), stage 3A (45-59 ml/min/1.73 m²), stage 3B (30-44 ml/min/1.73 m²), stage 4 (15-29 ml/min/1.73 m²), and finally stage 5 (<15 ml/min/1.73 m²) [11]. Data accumulated by the United States Renal Data System indicated that African American patients are three times more likely to develop end-stage renal CKD than white patients [9].

Management

The goal of managing patients with TRH caused by the progression of CKD is to monitor blood pressure and albuminuria. TRH is managed and achieved through multiple therapies, including ACE inhibitors, ARBs, CCBs, mineralocorticoid receptor blockers, and thiazide diuretics. The longer half-life of chlorthalidone and indapamide results in increased and potent natriuresis, which prevents blood pressure elevation. Furthermore, the restriction of dietary sodium intake by 1 g has shown a decrease in blood pressure by 2.1 mmHg, which would be beneficial in addition to the recommended therapy [12]. It is advised that blood pressure in TRH patients should be maintained at 130/80 mmHg or lower [13]. A study observed an increased utilization of ARBs, ACE inhibitors, CCBs, and thiazide-type diuretics in African American adults, ranging from 43.3% to 63.6% [13]. However, mineralocorticoids and diuretics of the thiazide-type are underutilized in African Americans despite their ability to lower blood pressure [13-14]. Agarwal et al. encouraged the use of Patiromer, a potassium binder combined with spironolactone for better blood pressure control and a decreased likelihood of hyperkalemia [14]. Arteriovenous coupling, baroreceptor activation therapy, renal artery stenting, and renal nerve denervation are experimental procedures that have been successful but still require extensive testing. Nonetheless, of all these procedures, only renal artery stenting is approved by the US Food and Drug Administration [7].

Renal artery stenosis and fibromuscular dysplasia

Renal artery stenosis (RAS) is known to be caused by fibromuscular dysplasia or atherosclerotic lesions [12]. Fibromuscular dysplasia is a rare non-inflammatory, non-atherosclerotic vascular disorder affecting multiple arteries, resulting in stenosis [15]. The prevalence of fibromuscular dysplasia is 0.4%, and it is seen more commonly in women between 20 and 50 years of age [12]. A study stated that men and African Americans with fibromuscular dysplasia were more likely to have atherosclerotic risk factors such as hypertension, diabetes mellitus, obesity, and CKD. African Americans are said to have a decreased prevalence of both fibromuscular dysplasia and renal artery stenosis [16]. Atherosclerotic causes of RAS are more prevalent in men and usually affect the aortic orifice or the proximal one-third of the renal artery. Furthermore, men and African Americans displayed increased susceptibility to atherosclerotic risk factors and vascular manifestations in comparison to women and Caucasians [16-17]. Although atherosclerotic lesions predominantly cause RAS, their etiology is not limited to the above-mentioned disorders. Other etiologies include nephroangiosclerosis, diabetic nephropathy, renal thromboembolic disease, thromboangiitis obliterans, and aortorenal dissection. In RAS, blood flow to the kidneys is distorted, which will inherently cause activation of the RAAS pathway, leading to hypertension [7].

Management

Maintaining target blood pressure (<130/80 mmHg) with minor medication adverse effects and minimal drug combinations is the main goal [13]. The use of ARBs and ACE inhibitors has shown a significant benefit in the mortality rate for managing resistant hypertension secondary to RAS [12]. ACE inhibitors or ARBs, in conjunction with beta-blockers, CCBs, and diuretics confer sufficient control on blood pressure [17]. Over nine months, revascularization decreased the mean systolic blood pressure from 162 mmHg to 145 mmHg in a non-randomized, single-arm study of 202 participants, but in comparison with medical therapy, eight trials with 2223 participants showed that revascularization produced no notable effect on systolic blood pressure [7]. The options available for revascularization are renal artery angioplasty and stenting, but the latter is associated with a better prognosis for avoiding restenosis [12,17]. Percutaneous angioplasty ranks higher than renal artery stenting in the treatment of fibromuscular dysplasia [12]. However, the indication for revascularization is ambiguous, leaving room for more studies to close the void of uncertainty [17].

Obstructive sleep apnea

OSA is defined as recurrent obstructive breathing events generated by complete upper airway obstruction during sleep and is accompanied by daytime symptoms and sleep hypoventilation syndrome [18]. Usually, patients complain of having witnessed apnea and loud snoring, frequent nocturnal awakening, feeling very tired during the day, fatigue, and low energy. The patient’s waking from sleep terminates the apneic and hypopneic episodes [19]. The oropharynx collapses during OSA events, causing arousal or oxygen desaturation, resulting in fragmented sleep [20].

OSA, a cause of high blood pressure, has also been associated with changes in day-to-night blood pressure, severe hypertension, and TRH [2]. Male sex is the strongest predictor of OSA, with symptoms exacerbated during supine and rapid eye movement (REM) sleep [21]. 

Sleep disturbances are prevalent among middle-aged and older adults and vary by race/ethnicity, sex, and obesity status [22]. The Sleep Heart Health Study found a slightly increased risk of moderate to severe OSA in African Americans (20%) and American Indians (23%) compared with whites (17%). The differences among racial groups may be due to variations in craniofacial anatomy [20]. According to an AHA journal on hypertension, a 70% to 90% prevalence rate of OSA has been reported among adult patients with TRH [2]. A study conducted on a population of African Americans living in the southern United States showed a high prevalence of moderate or severe OSA (24%), which was mainly undiagnosed (95%) [23]. Undiagnosed cases are particularly prevalent in African American patients [24].

Many pathophysiological mechanisms exist between hypertension and OSA despite several studies showing that the cause of OSA is mainly unknown. Negative intrathoracic pressure, which places stress on the heart, inflammatory and cytokine-mediated effects of hypoxia, impaired sleep quality, nocturnal fluid shift, increased sympathetic autonomic system, and decreased parasympathetic autonomic system lead to activation of the RAAS. These mechanisms potentiate TRH [19]. Studies have implicated excessive accumulation of fluid centrally, including the neck, as an essential contributor to OSA severity in patients with TRH by demonstrating an increased fluid shift from the lower extremities into the upper body during supine sleep [2].

Management

All patients with OSA should be assessed for treatment, including the exploration of possible behavioral modifications and weight loss. Appropriate diagnosis and management are required to prevent its harmful effects and death. Although not indicated for all patients with TRH, polysomnography is required when OSA poses a possibility of causing TRH. This requires the simultaneous monitoring of several cardiovascular and respiratory variables during nocturnal sleep (i.e., airflow, respiratory effort, oxygen saturation, sleep, and brain activity through electroencephalogram) [22]. Depending on the indication, behavioral modifications and weight reduction should be implemented when treating OSA [24]. Patients with TRH should be screened for symptoms of OSA, then assessed for their tolerance to continuous positive airway pressure (CPAP) before offering it to patients with severe OSA. When properly implemented, CPAP can be used for optimal treatment of OSA to maintain airway patency during sleep. It provides a relatively instantaneous relief of clinical symptoms, reduces the severity of OSA, and improves many acute and chronic pathophysiological alterations induced by OSA. Long-term use of CPAP shows marked and critical reductions in microneurography during sleep and wakefulness [22]. Several studies have also demonstrated the effectiveness of CPAP in improving baroreflex impairment, systemic inflammation, endothelial dysfunction, RAAS activation, arterial stiffness, and metabolic alterations. Patients with TRH who use CPAP regularly and for a long time have also shown a significant reduction in their daily and nighttime ambulatory blood pressure readings [22].

For patients who are unable to tolerate CPAP or those with mild to moderate OSA, oral appliances (OA) are available as an alternative [24]. OA are mandibular-repositioning appliances and tongue-retaining devices that enable protrusion and stabilization of the mandible to maintain a patent airway during sleep. These devices are available as custom or non-custom types. Depending on the type, OA has improved daytime sleepiness, oxygen saturation, sleep hygiene, and quality of life [25]. They are considered second-line therapy in OSA treatment [26]. Although some patients have reported favorable effects of OA on blood pressure levels, more evidence is needed to confirm its effectiveness in TRH [22]. A meta-analysis of seven studies (399 OSA patients involved) found that treatment with OA was more beneficial for blood pressure reduction than CPAP therapy [19]. A follow-up assessment should determine the effectiveness of OSA treatment in patients with TRH.

Samuelson et al. described a surgical technique in OSA management called uvulopalatopharyngoplasty (UPPP) [26]. UPPP is the most common surgical procedure for OSA to minimize the frequency of nocturia in patients with mild-to-moderate OSA. UPPP removes excess tissue from the soft palate and pharynx to reduce obstruction that causes OSA in many patients.

A list of other surgical procedures that have significant positive outcomes in the management of OSA includes hypoglossal nerve stimulation, hypopharyngeal procedure, soft palate implant, tracheostomy, maxillomandibular advancement (MMA), uvulopalatine flap, and tonsillectomy in adults with tonsillar hypertrophy [26].

Endocrine causes of TRH

Primary Hyperaldosteronism (Conn's Syndrome)

The use of diagnostic thresholds coupled with specific laboratory biochemical cutoffs (e.g., aldosterone: renin ratio; normal values: 0-30 ng/dL per ng/mL/hour) often aids in diagnosing primary hyperaldosteronism. It is defined as an aberration from normal physiology in which aldosterone is produced independently of its dominant regulators, resulting in a shift from normovolemia. The initial insult often involves the formation of one or more aldosterone-secreting tumors in either a unilateral or bilateral manner. This secretion is independent of the RAAS and also results in a hypokalemic state, which physiologically should suppress aldosterone secretion. The net effect of this pathophysiologic process is to increase sodium and water reabsorption. Subsequently, an isotonic volume expansion occurs, which enhances glomerular hyperfiltration and sets in motion a vicious cycle of distal sodium delivery. The net effect is increased sodium reabsorption and hypervolemia [27]. Approximately 5-12% of patients with hypertension have Conn's syndrome [28]. Amongst patients with TRH, about 22% have primary hyperaldosteronism [29]. A higher prevalence of TRH consequences, such as heart failure and death, among African Americans, is attributed to increased RAAS activation in hyperaldosteronism. Therefore, it is suggested that the RAAS be used to identify high-risk people to improve therapeutic and preventive methods for cardiovascular-related mortality in African Americans [30].

Management: Surgical adrenalectomy is the best option for long-term blood pressure management in patients with unilateral adrenal lesions as it has curative intent. Several indicators that predict a favorable outcome of surgical adrenalectomy have recently been established. These include: a duration of hypertension of fewer than five years; treatment with two or fewer antihypertensive agents, preoperative response to spironolactone, and the underlying lesion being an adenoma rather than adrenal hyperplasia. The primary aldosteronism surgery outcomes (PASO) study demonstrates that most patients with unilateral Conn syndrome have persistent hypertension, although in a significantly milder form. However, curative unilateral adrenalectomy has proven to be instrumental in preventing incidental atrial fibrillation compared to medical therapy with spironolactone. Recently, venous sampling approaches that pinpoint the exact location of aldosterone production within the adrenal gland have heralded promising supplementary surgical options. Partial adrenalectomy has been employed in both unilateral and bilateral primary hyperaldosteronism and yielded results pointing towards future success. Nevertheless, the technique has not had extensive clinical implementation [29,31].

Spironolactone is a practical, affordable, and readily available treatment for primary hyperaldosteronism in most individuals. However, there are concerns over long-term outcomes with mineralocorticoid antagonists, such as hyperkalemia, bleeding after menopause, and diarrhea. Thus, surgery remains the ideal option for eligible candidates. Medical therapy with spironolactone or eplerenone coupled with restriction of dietary sodium is recommended if the patient is not a surgical candidate or has bilateral disease. Patients with Conn syndrome usually receive 200 mg of spironolactone daily, resulting in significant reductions in cardiovascular events. Optimal response to mineralocorticoids may be achieved with additional medications such as patiromer and amiloride [31-32].

Pheochromocytoma

This clinical entity involves chromaffin cell tumors of the adrenal medulla that result in a supranormal secretion of catecholamines. Pheochromocytomas occur sporadically or are usually associated with other familial endocrinopathies or syndromes (e.g., multiple endocrine neoplasia), in which case the disease usually has a bilateral presentation. The high levels of circulating hormones result in an increased adrenergic response of the cardiovascular system, thereby leading to hypertension. Hypertension in pheochromocytoma often manifests in an episodic pattern accompanied by headaches and sweating. In between episodes, patients are often normotensive. The disease poses a more significant challenge to individuals with a pre-existing diagnosis of hypertension, as this may result in TRH [33]. The prevalence is relatively low, pegged at 0.01% to 0.2%. Initial screening is achieved by measuring circulating catecholamine metabolites in serum or urine.

Management: Imaging using an abdominal MRI or CT scan confirms the diagnosis, which usually warrants surgical treatment with unilateral adrenalectomy. To minimize intraoperative blood pressure instability and counter volume contraction from pressure natriuresis, alpha-adrenergic blockade using agents like phenoxybenzamine, increased fluid intake, and a high sodium diet are recommended for at least a week before surgery. Post-surgical follow-up is of the essence in patients with a familial form of the disease [2].

In metastatic disease, combination chemotherapy with cyclophosphamide, vincristine, and dacarbazine (CVD) is associated with clinically beneficial outcomes. This is echoed by the results of a multivariate study that demonstrated a significant median overall prolonged survival among those who received CVD (p = 0.05; hazard ratio = 0.22; 95 % confidence interval = 0.05-1.0) compared to other combinations [34]. Radiopharmaceuticals such as 131I-metaiodobenzylguanidine (MIBG), peptide receptor radionuclide therapy (PRRT), and immunotherapy with pembrolizumab also provide great alternative treatment options in individuals with metastatic disease [34].

Hypercortisolism (Cushing Syndrome)

Cushing syndrome is an endocrinopathy involving chronic excessive exposure to high levels of endogenous or exogenous glucocorticoids, with the latter accounting for most cases. Endogenous causes are further classified into adrenocorticotropic hormone (ACTH) dependent (ACTH-secreting pituitary adenomas and ectopic ACTH-secreting neoplasms) and ACTH-independent (adrenal hyperplasia, carcinoma, and adenoma).

There is high variability in the prevalence of Cushing syndrome amongst different ethnic groups. According to studies, up to 80% of cases of hypercortisolism are due to iatrogenic causes [35]. Besides the more prominent effects of Cushing syndrome, such as metabolic derangements (increased insulin resistance, hyperglycemia) and immunosuppression, the permissive effect of cortisol on catecholamines results in elevated blood pressure. This ultimately results in TRH, particularly in patients with baseline hypertension. As such, treatment considerations must be made once the diagnosis is confirmed.

Management: Tapering exogenous corticosteroids provides the basis of treatment for iatrogenic causes of Cushing syndrome. If done appropriately, outcomes are often favorable with a return to physiologic hormone levels. Individuals diagnosed with adrenal tumors, pituitary adenomas, or ACTH-secreting neoplasms usually benefit from surgical resection. Importantly, post-surgical follow-up with measurement of free urine or serum cortisol to monitor recurrence should be performed. In patients with pituitary adenomas that fail to respond to transsphenoidal resection, radiotherapy yielded desirable outcomes. Poor surgical candidates or those that preferred conservative treatment options benefited from oral medications like cabergoline, pasireotide, and mifepristone [35].

Other rare endocrine causes of TRH

The literature describes other uncommon endocrine diseases that account for a negligibly small part of TRH prevalence. Table 1 summarizes some of these causes.

**Table 1:** Other rare endocrine causes of TRH ^a^Despite appropriate treatment of acromegaly, it has been reported that hypertension often resolves only partially [36-37]. ^b^A racial predilection has been reported among African Americans [38]. ^c^The color of one's skin influences the amount of 25(OH)D in the blood, and African Americans are known to have much greater rates of hypertension than whites [38].

Cause	Prevalence	Pathophysiology	Management
Acromegaly	2-11 people per million annually^a^	Chronic excessive exposure to high levels of growth hormone and insulin like growth factor 1	Surgery dopamine agonists somatostatin receptor ligand Pegvisomant^a^
Hyperthyroidism	In the US, overall: 1.2%, overt: 0.5%, subclinical: 0.7%^b^	Excessive thyroid hormones in tissues caused by: Graves disease, thyroid adenoma, pituitary adenoma, factitious thyroiditis	Surgery thyroidectomy + hormone supplementation, antithyroid medications methimazole propylthiouracil radioiodine beta blockers
Vitamin D deficiency and Hyperparathyroidism	In the US, 0.86% of the population^b^	Activation of the RAAS parathyroid hormone secreting tumors or hyperplasia^c^	Vitamin D and calcium supplementation, surgical resection of adenomas, cinacalcet, bisphosphonates

Sympathetic nervous system overactivity

Human SNS dysfunction is linked to the onset and progression of hypertension, heart failure, and chronic kidney disease and is a major player in sustaining TRH. SNS overactivity as a cause of TRH is characterized by overstimulation of the RAAS with subsequent sodium retention [39]. This leaves a trail of vascular disease or dysfunction as demonstrated by increased rates of peripheral and carotid artery atherosclerosis, decreased endothelial function, lower arterial compliance, and elevated systemic vascular resistance, all of which may be more severe in TRH patients than in non-TRH patients. In most people with TRH (43-65%), the typical nocturnal drop in blood pressure is less significant due to SNS overactivity [2].

Management

SNS overstimulation is managed by device-based treatment, which includes renal sympathetic denervation and baroreflex activation therapy. Renal sympathetic denervation is a non-pharmacological method of TRH treatment. In this regard, catheter-based devices can be employed to ablate the renal artery's sympathetic afferent and efferent nerves by radiofrequency or ultrasound energy or by transarterial injection of acidic substances [40]. An alternate pathway to decreasing sympathetic tone to help control blood pressure is electric stimulation or ablation of the carotid sinus baroreceptors. Some advantages of this procedure are that it attenuates overall sympathetic outflow and it has the potential for neurohormonal modulation. Nevertheless, it has some limitations, including the need for subcutaneous internal pulse generation with some systems, different responses to carotid sinus stimulation, surgical neck dissection, and the risk of nerve injury with an associated residual deficit [41].

Lifestyle factors

Evidence-based studies have proven a relationship between lifestyle factors and hypertension. These lifestyle factors include but are not limited to obesity, physical inactivity, dietary habits, sleep deprivation, alcoholism, and drugs, which in the long term can lead to TRH. Of note, behavioral factors tend to be more prevalent in African Americans than in Asians or whites [2].

Obesity

Some cases of uncontrolled blood pressure and TRH in African American adults have been linked to obesity. Obesity continues to rank as one of the most critical public health problems in the USA, with nearly two-thirds of US adults (66.3%) considered to be overweight (BMI 25-29.9 kg/m²; 34.1%) or obese (BMI ≥ 30 kg/m²; 32.2%) [42]. While lifestyle interventions can improve blood pressure, weight loss improves metabolism, stabilizes neurohormonal activity, and causes clinically significant reductions in blood pressure. Visceral adiposity plays a pivotal role in causing high blood pressure through various mechanisms, resulting in enhanced salt sensitivity, vascular dysfunction, increased mineralocorticoid activity, activation of the SNS, and RAAS [2].

Dietary Habits

According to several studies, there is evidence to suggest that African Americans are more salt-sensitive than Caucasians, which is due to a tendency to retain sodium in the kidneys [43]. Salt sensitivity appears more prevalent in the elderly, black people, and people with metabolic syndrome or obesity. Irrespective of sex and ethnic group, a modest reduction in dietary sodium intake induces a significant fall in blood pressure in hypertensive and normotensive individuals, with a more evident decrease in systolic blood pressure [44]. The dietary approach to stop hypertension (DASH) diet can lower blood pressure in proportion to the severity of hypertension. In a recent meta-analysis, a DASH diet was well established to reduce blood pressure by 6.7/3.5 mmHg, although the diet is not independently associated with TRH [2].

Sleep Deprivation

Growing evidence has implicated sleep deprivation or poor sleep hygiene as one of several risk factors that are thought to contribute to high blood pressure or TRH in the African American population. The Center for Disease Control and Prevention (CDC) has recommended a seven-hour sleep duration for adults per night for good health and well-being. Short sleep duration is defined as less than seven hours of sleep per 24-hour period. Adults who were short sleepers (less than seven hours per 24-hour period) were more likely to report 10 chronic health conditions than those who got enough sleep (seven or more hours per 24-hour period). Short (<6 hours) and very long (≥9 hours) sleep duration has been associated with poorer cardiovascular health compared with sleep lasting seven to eight hours [45].

Physical Inactivity

In the USA, older African Americans report high levels of physical inactivity, especially those with chronic conditions such as hypertension, diabetes mellitus, arthritis, and chronic obstructive pulmonary disease (COPD) [46]. Physical activity is recognized as an essential component of a healthy lifestyle. Previous studies have shown that many adults may find it challenging to undertake exercise that fits into their daily lives. Walking is a low-cost activity that many people can do. A randomized study demonstrated that a thrice-weekly treadmill walking exercise program for eight to 12 weeks significantly lowered daytime ambulatory blood pressure (6±12/3±7 mmHg; P = 0.03) among 50 treated patients with TRH [2].

Tobacco Smoking and Alcoholism

Smoking, a modifiable risk factor for hypertension, can contribute to the development of atheromatous RAS, a leading cause of TRH [47]. Notably, non-selective beta-blockers are not as effective in smokers as in nonsmokers, which can also be a factor in TRH [48]. Physicians should make an effort to counsel patients on smoking cessation regardless of the patient's age and length of tobacco use. Various smoking deterrents can help some patients quit. Smoking is often associated with increased alcohol consumption [47]. A study conducted among economically disadvantaged African American older adults showed that smoking and alcoholism are more common among the African American population. This study blamed their vulnerability on financial difficulty, educational attainment, employment, and a reduced likelihood of access to cessation coping mechanisms. The pattern and predictors of smoking in the African American community may differ from those in other communities. The undesired health outcomes associated with smoking and alcohol use are more common in the African American population. They are believed to result in more cancer, cardiovascular, liver, and respiratory diseases in African Americans than whites [49]. Heavy alcohol use (>30-50 g/dl) doubles the risk of hypertension. Additionally, it has been reported to worsen other conditions predisposing to TRH, such as OSA [21,48-47]. A study has shown that while varenicline alone may be sufficient for smoking cessation in heavy-drinking smokers, administering varenicline plus naltrexone may confer benefits concerning drinking outcomes, especially during the 12 weeks of active medication treatment [50].

Drugs

Several studies have described various drugs that increase blood pressure significantly. However, some effects may be reversible with body adjustments or discontinuation. A few others may have contributed to the development of TRH. The implicated pharmacological agents include non-steroidal anti-inflammatory drugs (NSAIDS), erythropoietin, vascular endothelial growth factor (VEGF) inhibitors, sympathomimetics, oral contraceptives, alcohol, amphetamines, antidepressants, cocaine, glucocorticoids, mineralocorticoids, and immunosuppressive agents [2].

Suboptimal treatment

A clinician-centered factor contributing to poorly controlled blood pressure levels is that patients are not receiving the proper treatment regimen. An article published by AHA reports that black adults with TRH are either inappropriately treated or lack appropriate counseling about healthy lifestyles and behavioral practices to lower blood pressure. Carey et al. documented a low use of antihypertensive medications and poor lifestyle factors among African Americans with apparent TRH. Previous studies show that regulating blood pressure according to the 2017 American College of Cardiology/AHA blood pressure guideline could prevent about 400,000 CVD events per annum in African Americans and that the population-attributable risk of hypertension is higher in black compared to white adults. Of note, blood pressure control is more challenging in black adults than in white adults [2]. Therefore, achieving higher rates of blood pressure control among black adults may be essential in reducing disparities in hypertension-related morbidity and mortality. The use of CCBs, ACE inhibitors, and ARBs among black adults with apparent TRH was relatively high, ranging from 43.3% to 63.6%, as described by Langford et al. [13]. Nonetheless, the use of thiazide-like diuretics and mineralocorticoid receptor antagonists was low. These observations resonate with findings from a study by Fontil et al. that looked at the underutilization of the most effective medications for TRH using 2006 to 2010 data from the National Ambulatory Medical Care Survey (N=1567; n=313 Blacks). Among patients with apparent TRH, chlorthalidone and a mineralocorticoid receptor antagonist were taken by <3% and <4%, respectively. The underutilization of thiazide-like diuretics and mineralocorticoid receptor antagonists in patients with apparent TRH shows a missed opportunity to adequately achieve blood pressure control [13].

Medication adherence is another crucial factor in hypertension management. Unfortunately, one-quarter of patients who start antihypertensive treatment do not complete their initial prescription, and 50% to 80% do not adhere to their medications properly. The significant pill burden, dosing complexity, expense, high frequency of adverse reactions, poor patient-clinician relationship, and clinician inertia with reduced insistence on adherence are all factors that contribute to this relatively high proportion of nonadherence that may mimic TRH [2,51]. A study reported at the AHA's Joint Hypertension 2018 Scientific Sessions points out that approximately one in every five patients does not achieve target blood pressure simply because they "do not take their pills." This often poses a challenge in determining the true prevalence of TRH [2]. Frank and nonjudgmental clinician-patient dialogue, monitoring of prescription refills and pill counts, and, if possible, biochemical analysis of medications or their metabolites in urine or plasma should all be used to rule out nonadherence [40].

Inaccurate measurement

The misinterpretation of TRH may be due to blood pressure measurement errors. This arises when blood pressure measurement is not done according to recommended guidelines and can result in apparent TRH. The accuracy rate of blood pressure measurements was found to be 33% in a study of patients referred for TRH [2]. Patient preparation, environmental conditions, cuff size, and measurement technique can significantly impact blood pressure findings. Because of the inherent blood pressure fluctuation, diagnostic blood pressure recordings must contain an average of at least two readings taken on at least two different occasions. Similarly, a suitable approach is required for out-of-office and self-monitored blood pressure. Another cause of inappropriate measurement occurs in a subset of patients with white-coat hypertension whose CVD risk is similar to that of hypertensive patients with well-controlled blood pressure. Therefore, out-of-office blood pressure monitoring is usually required to diagnose genuine TRH. This can be achieved by the use of a 24-hour ambulatory blood pressure monitor (ABPM), which can easily detect the white-coat effect [51].

## Conclusions

The treatment success of TRH depends on an extensive workup of probable underlying causes that requires an individualized management approach with prompt interventions resulting in better outcomes. Remarkably, the true prevalence of TRH in African Americans can be limited by intensifying emphasis on addressing modifiable risk factors such as lifestyle behaviors, administration of appropriate antihypertensive medications, and treatment compliance and adherence. Constant communication and follow-up amongst multidisciplinary team members are equally essential to facilitate maximal healthcare delivery, minimize the prevalence of TRH and other cardiovascular implications, improve quality of life, and reduce mortality risk. Research gaps in the African American subpopulation indicate the need for more extensive study of this clinical entity in the respective category.
